# Multiple myeloma with secondary amyloidosis: Dysphagia as the first symptom: A case report

**DOI:** 10.1097/MD.0000000000038968

**Published:** 2024-07-12

**Authors:** Bing Xue, Liang Li, Shanshan Ma

**Affiliations:** a Department of Geriatrics, Zibo Central Hospital of Shandong University, Zibo, Shandong, China; b Department of Gastrointestinal Surgery, Zibo Central Hospital of Shandong University, Zibo, Shandong, China.

**Keywords:** amyloidosis, dysphagia, hoarseness, multiple myeloma

## Abstract

**Rationale:**

Multiple myeloma (MM) with secondary amyloidosis (AL) is a rare clonal plasma cell proliferation disease, which causes dysfunction of multiple organs and tissues. We report a case of dysphagia as the first symptom in a patient with MM and secondary AL.

**Patient concerns:**

The patient was a 73-year-old female, was admitted to our hospital, because of progressive dysphagia for 4 months and limb weakness for 1 month.

**Diagnoses:**

The bone marrow smear and pathology diagnosis revealed the presence of MM, while the biceps myopathy diagnosis indicated AL.

**Interventions:**

The VCD regimen consisted of bortezomib at a dosage of 1.9 mg on days 1, 8, 15, and 22, cyclophosphamide 0.4 g on days 1, 8, and 15, and dexamethasone at a dosage of 40 mg on days 1, 8, 15, and 22. The patient simultaneously received comprehensive treatment including anti-infective therapy, enhanced cardiac function, and nutritional support.

**Outcomes:**

The M protein in the blood and urine protein were negative, indicating a reduction in bone marrow plasma cells to 2%. Flow cytometric analysis revealed a minimal percentage 0.04%. As a result, complete remission was achieved.

**Lessons:**

The clinical manifestations of MM exhibit a wide range, with the symptoms of secondary injury causing significant disturbing, while the atypical symptoms of extramedullary manifestations pose challenges in diagnosing the disease.

## 1. Introduction

Multiple myeloma (MM) is a malignant tumor characterized by the clonal proliferation of malignant plasma cells in the bone marrow. It is not only present in the bone marrow but also in almost any organ system outside the marrow. Extramedullary disease is thought to be associated with hematogenous spread, which allows cells to escape from the bone marrow when the expression of receptors on the surface of myeloma cells is reduced. Amyloidosis (AL) is a systemic disease in which monoclonal immunoglobulins are deposited in organ tissues and cause abnormalities in the function of the corresponding organ tissues. We treated a rare case of extramedullary MM with secondary AL, and this article reviews the diagnostic process of this patient and incorporates a literature review to increase clinicians’ awareness of this disease.

## 2. Case presentation

A 73-year-old female patient was admitted to our Department of Neurology of hospital on July 12, 2023, due to progressive dysphagia for 4 months and weakness of limbs for 1 month as the chief complaint. The patient presented with dysphagia 4 months ago, with progressive aggravation, gastroscopy showed chronic non-atrophic gastritis. 2 months before the onset of unclear articulation and hoarseness. 1 month ago, the patient had weak limbs, difficulty lifting heavy objects with both upper arms, and difficulty in squatting and climbing stairs with both lower limbs. At the same time, drinking water cough appeared. Electronic pharyngoscopy showed that saliva retention was found in the bilateral piriform fossa, and other abnormalities were not found. 9 days ago, she was basically unable to eat water through her mouth. She went to the emergency department of our hospital to indenture a gastric tube and was then fed nasally. The patient began to lose more than 20 kilograms of weight. The previous gastroesophageal reflux was nearly 1 year and worsened for 4 months. She underwent lumbar surgery for lumbar disc herniation 10 years ago and cervical surgery for cervical disc herniation 6 years ago.

Emaciation, slurred speech, hoarse voice, discernible speech content. The muscle volume of the tongue and throat muscles was normal. Bilateral soft palate lift is weak, uvula is centered. No atrophy of tongue muscle, no fibrillation, limited tongue extension, weak tongue against cheek. The bilateral pharyngeal reflex is present. No muscular atrophy was observed in the limbs, and the muscle tone in the limbs was normal. Double upper limb muscle strength grade 4, double lower limb proximal muscle strength grade 4, distal muscle strength grade 5, fatigue test negative. Deep and shallow sensory examination showed no abnormalities. Active tendon reflexes in the extremities, bilateral Pap positive. No abnormal gait was observed. Meningeal stimulation is negative. Mild concave edema of both lower limbs.

### 2.1. Laboratory analysis result

Plasma D-dimer 1.12 µg/mL (normal reference values 0–0.5, the same below), white blood cell count 10.23 × 10^^9^/L (4–10), total protein 56.57 g/L (60–80), albumin 34.05 g/L (40–60), prealbumin 75 mg/L (280–360), immunoglobulin M 0.32 g/L (0.6–2.5), complement C3 0.67g/L (0.85–1.7), urine protein 2+, NT-proBNP 961.00 pg/mL (<100). 24h urine albumin is quantitatively 2.67g/24h (100–150 mg). Blood myasthenia gravis antibody profiles were all negative.

### 2.2. Imaging analysis results

Cardiac ultrasound indicated main and pulmonary regurgitation (mild), mitral valve prolapse with regurgitation (mild), tricuspid valve regurgitation (mild-moderate), and left ventricular diastolic insufficiency. Deep vein ultrasound of both lower limbs showed no obvious abnormality. Head magnetic resonance imaging (MRI) plain scan showed no abnormal changes in the brain, cervical spine MRI showed postoperative changes in the 4 to 7 vertebrae of the neck and cervical degenerative changes. Cervical 3 to 4 disc distension, narrow spinal canal; Abnormal signals in the spinal cord at the level of the cervical 5 vertebrae, ischemic changes are possible. MRI plain scan of the thoracic vertebra revealed degenerative changes in the thoracic vertebra. Abnormal signal of thoracic 4 vertebrae; Thoracic 9 vertebral level right nerve root sheath cyst. FDG-PET-computed tomography (CT) showed a moderate decrease in glucose metabolism in the local cortex of bilateral frontal, parietal and left temporal lobes, double lung infection, enlargement of lymph nodes in 4 mediastinal groups, and slightly higher metabolism.

### 2.3. Other inspections and analyses result

Neostigmine test: The symptoms of dysphagia, hoarseness, and limb weakness were observed after 1mg of neostigmine was intramuscularly injected. The patients’ symptoms were evaluated every 10 minutes after intramuscular injection and kept for 60 minutes. The cerebrospinal fluid pressure was 75 mmH_2_O, and the routine cell biochemistry of the cerebrospinal fluid was normal. Blood and cerebrospinal fluid para-tumor syndrome antibody spectrum, autoimmune encephalitis antibody spectrum, and demyelinating antibody spectrum were negative. Electrophysiological examination showed that the left deltoid muscle, left abductor pollicis breve, right quadriceps muscle, right sternocleidomastoid muscle, and left tongue muscle showed myogenic damage, and the right anterior tibial muscle showed neurogenic damage. The low frequency (3HZ, 5HZ) of the right facial nerve, right accessory nerve and right ulnar nerve, and the high frequency (30HZ) of the right ulnar nerve did not increase or decrease.

The patient had cough and sputum upon admission; lung CT indicated inflammatory changes, transient hypothermia and hypoxemia, progressive decrease of hemoglobin and albumin, and progressive increase of NT-proBNP, complicated by pleural effusion and ascites. After multidisciplinary diagnosis and treatment, the patient was given comprehensive treatment such as anti-infection, relieving asthma, reducing phlegm, improving cardiac function and nutritional support. According to the qualitative diagnosis of the primary disease, relevant examinations were improved, and the possibility of neuromuscular junction disease, motor neuron disease, tumor-related diseases, polymyositis, vasogenic diseases, genetic metabolic diseases, and autoimmune encephalitis were excluded. Since the electromyography indicated myogenic injury, the muscle MR, muscle biopsy, and anti-myositis antibody spectrum examination of the limbs were improved. MRI plain scan of both thighs revealed abnormal signals in the right medial femoris muscle and edema signals in the subcutaneous fat space of both thighs. MRI plain scan of the left upper arm indicated abnormal signals of the left deltoid and biceps. The anti-myositis antibody profile showed positive anti-MI-2. HE staining of the biceps muscle tissue showed abnormal material deposition around some muscle fibers and no inflammatory cell infiltration. Congo Red stain (+). The pathological diagnosis of the muscle was amyloid myopathy.

**Figure 1.**
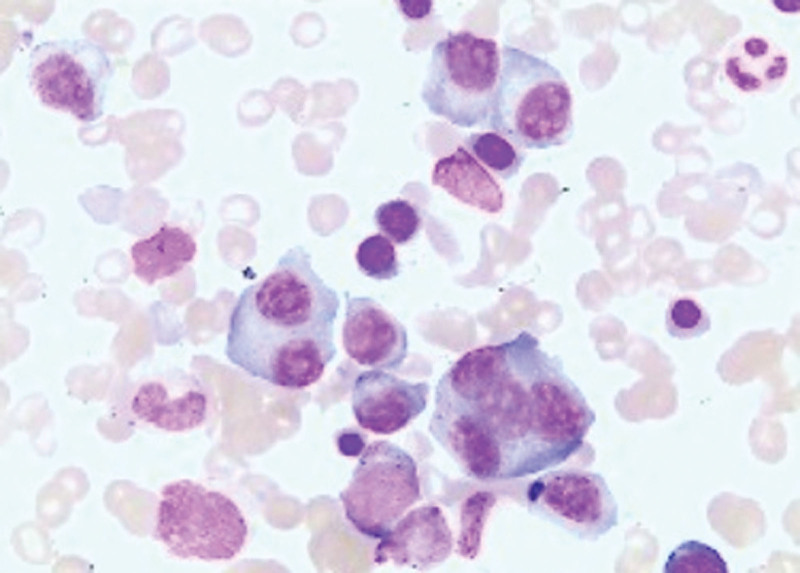
Bone marrow smear (100×): 18% plasma cells, visible as primary and young plasma, binucleated and multinucleated plasma cells.

**Figure 2.**
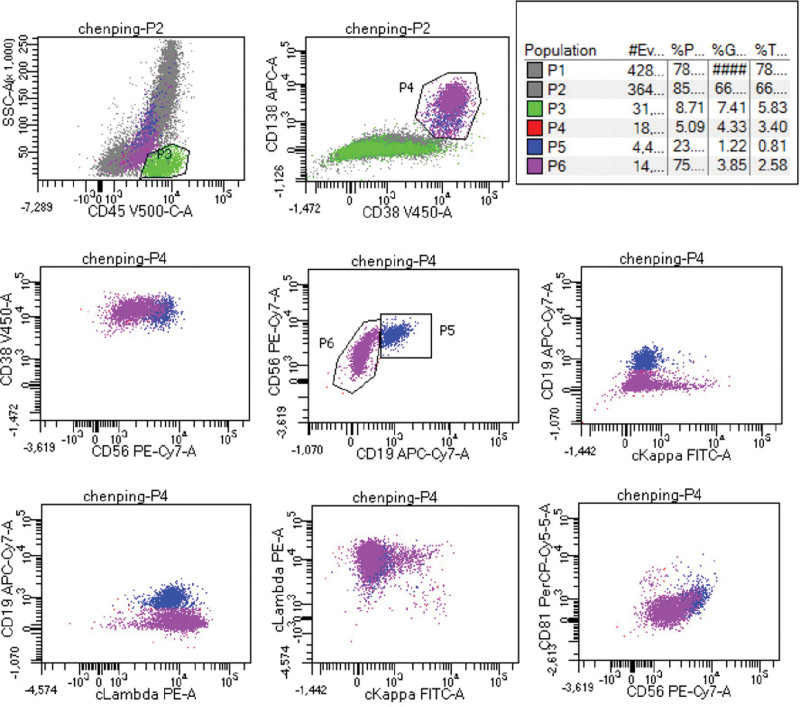
Bone marrow flow cytology: P5 and P6 are both bone marrow monoclonal plasma cells.

At present, systemic AL and inflammatory myopathy cannot be ruled out by qualitative diagnosis. Intravenous infusion of gamma globulin has not significantly improved the symptoms of patients. According to proteinuria, anemia, and polyserous effusion, secondary systemic AL was first considered, and blood and urine light chain protein and immunofixation electrophoresis were further improved. And perfect bone marrow puncture examination. Urine free light chain κ13.08 mg/L, λ4255.79 mg/L, serum-free light chain κ18.59 mg/L, λ1991.44 mg/L, light chain λ-M protein appeared in urine electrophoresis, urine immunofixation electrophoresis positive. Serum immunofixation electrophoresis showed the presence of light chain λ M protein. Bone marrow puncture (Fig. 1): Plasma cells accounted for 18% of the bone marrow cell morphology, and protoplasma, juvenile plasma, binucleated and multinucleated plasma cells were visible. Bone marrow flow cytometry (Fig. 2) showed that P5 accounted for 1.22% of nucleated cells in bone marrow and expressed CD45dim + CD38++CD138++CD19 + CD56++ CD81-Cappa-cLambda+. P6 accounted for 3.85% of bone marrow nucleated cells and expressed CD45dim + CD38++CD138++CD19-CD56 + CD81-Cappa-cLambda+. P5 and P6 are monoclonal plasma cells. Bone marrow FISH was positive for TP53 deletion and IGH rearrangement.

The patient was eventually diagnosed with MM (light chain λ type) ISS stage III TP53 deletion, positive IGH rearrangement, and secondary AL. After hospitalization, the patient developed pulmonary infection, heart failure, pleural effusion, ascites, and pericardial effusion, and the symptoms improved after multidisciplinary consultation and treatment. The primary disease was treated with VCD chemotherapy (bortezomib 1.9mg,d1, d8, d15, d22; cyclophosphamide 0.4g, d1, d8, d15; dexamethasone 40mg, d1, d8, d15, d22). After 2 courses of treatment, the blood M protein and urine protein turned negative, 2% of bone marrow plasma cells were found, 0.04% could be seen by flow cytometry, and the disease achieved complete remission. The dysphagia of the patient did not improve significantly, and the third course of treatment was adjusted to the D-VD regimen (bortezomib 1.9mg,d1, d8, d15, d22; dexamethasone 40mg d1, d8, d15, d22; daretuzumab 700mg/d,d1, d8). Four courses of the D-VD regimen have been completed, and the patient condition has improved, and follow-up treatment is continuing.

## 3. Discussion

The abnormal proliferation of monoclonal plasma cells in the bone marrow and the production of monoclonal immunoglobulin lead to the injury of related organs or tissues in patients with MM. Studies show that almost all MM cases were previously accompanied by monoclonal gammaglobulinopathy of unknown significance, an asymptomatic nonmalignant disease confined to bone marrow and bone.^[1]^ Extrameductary multiple myeloma (EM) can involve any organ in the body and accounts for 10%-16% of myeloma cases,^[2]^ with a higher proportion of men and women than typical MM.^[1,3]^ When only extramedullary soft tissue is involved, it is called extramedullary plasmacytoma (EMP). In 80% of cases, EMP involves the upper respiratory digestive tract,^[4]^ and gastrointestinal tract involvement has also been reported.^[5]^ According to Bladé,^[6]^ EMP may have 2 different origins: direct mechanical extension from the bone marrow when EM destroys cortical bone and spreads to adjacent soft tissues, or blood-borne metastatic spread when reduced adhesion receptors on selected cell surfaces allow bone marrow release. The typical features of this patient lacking MM include bone pain, anemia, and hypercalcemia, and the main manifestations of extramedullary myeloma are dysphagia, slurred speech, and weak limb muscle strength. According to the diagnostic criteria for MM updated by the International Myeloma Working Group,^[7]^ MM can be diagnosed through bone marrow examination.

AL is the fibrous aggregate of the light chain of monoclonal immunoglobulin, which is deposited in various organs such as the liver, heart, kidney, and gastrointestinal tract, resulting in sexual organ dysfunction and death.^[8]^ Accumulation of amyloid deposits in multiple organs predicts a poorer prognosis and different treatment compared to amyloid deposits in a single organ.^[9,10]^ Despite impressive advances in the treatment of MM, MM-related AL is also considered an independent high-risk prognostic factor in patients with MM, even when there are no symptoms at the time of diagnosis. The heart is the most commonly affected organ, with cardiomyopathy occurring in 70% of AL patients. Patients with heart failure caused by amyloid deposits in the heart have a much shorter survival time than those without heart failure. Therefore, the presence and degree of cardiac involvement are key determinants of patient prognosis.^[11]^ MM and AL share a common etiological root, that is, the presence of clonal malignant plasma cells in the bone marrow, which behave differently, leading to significantly different clinical outcomes. Both may exist at the same time or start with one and then evolve into the other. The 2 can be mutual pathogenesis factors, but so far, the pathogenesis and mutual secondary relationship between MM and AL are not clear, and the prognosis is not good.^[7,12]^

The clinical manifestations of MM are varied and nonspecific. Macroglossus is a rarely reported chief complaint in extramedullary MM.^[13]^ Dysphagia is a rare manifestation of gastrointestinal AL, and common symptoms of esophageal involvement are dysphagia, chest pain, heartburn, and hematemesis. It has been reported that patients with GI involvement have a worse prognosis than those without GI involvement.^[14]^ This patient is a rare case of MM with light chain AL. The tongue is not obviously involved, but the throat and esophagus are involved, and the first symptom is dysphagia, followed by hoarseness. These sites are not common sites for amyloid deposition. Multidisciplinary joint diagnosis and treatment should be discussed to avoid missed diagnosis and misdiagnosis, and the best treatment measures should be given to patients in time.

Clinical, pathological, and radiological measures are used in the diagnosis of MM. The patient had no typical manifestations of MM, mainly secondary symptoms, so the initial diagnosis was in the Department of Neurology, and the diagnosis and treatment process was complicated, which brought difficulties to the final diagnosis. Cytology and flow cytometry can show monoclonal cell expansion, but the initial diagnosis in most patients is difficult and often requires a definitive diagnosis during a patient relapse or disease progression.

At present, the treatment and prognosis of MM with AL are not ideal. The therapeutic goal of AL is to reduce the level of monoclonal immunoglobulin light chain in the body, prevent further deposition of amyloid protein in important organs, and alleviate or reverse organ dysfunction caused by amyloid deposition. The main therapeutic method is to remove plasma cell cloning that produces abnormal light chains. Merlini emphasizes that the treatment of light AL should be highly individualized according to age, organ dysfunction, and regimen toxicity, and should be guided by biomarkers of hematology and cardiac response.^[15]^ Bortezomib-based treatment regiments have been shown to provide better response rates and survival times than traditional chemotherapy regiments,^[16]^ and hematopoietic stem cell transplantation has become an important treatment mode.^[15]^ Immunotherapy targeting amyloid deposits has been reported to help reverse organ dysfunction.^[17]^ Oliver-Caldes first reported the treatment of AL and relapsed/refractory MM using chimeric antigen receptor T cells (CART) of second-generation B cell maturation antigen (BCMA), and the results showed that patients with MM and AL could benefit from CART treatment.^[18]^ However, in patients with heart or kidney dysfunction, immune-related toxicity is not tolerated.^[19]^ Since there is no cure for MM to date, lifelong tumor follow-up is critical. Studies have shown that minimal residual lesions (MRD) in bone marrow can more sensitively and deeply reflect the depth of disease remission, and is closely related to the prognosis of the disease.^[20]^ The study of Rios-Tamayo showed that NT-proBNP ≥ 8500 pg/mL was an independent prognostic factor for overall survival, while cardiac ejection fraction had a marginal effect.^[21]^ In addition, age is also a very strong prognostic factor for AL and MM. Patients with MM and AL have a poor prognosis, and patients often die from cardiac AL, renal failure, and gastrointestinal bleeding caused by AL, rather than MM-related death.

## 4. Conclusions

The clinical manifestations of MM exhibit a wide range of diversity, and the occurrence of secondary AL-induced damage is infrequent. Consequently, diagnosing this disease in patients presenting with dysphagia as the first symptom poses a significant challenge. Early diagnosis of such diseases is particularly important, as treatment aimed at preserving organ function can potentially enhance patient prognosis.

## Author contributions

**Conceptualization:** Bing Xue.

**Data curation:** Bing Xue, Liang Li.

**Formal analysis:** Bing Xue.

**Investigation:** Bing Xue, Shanshan Ma.

**Supervision:** Shanshan Ma.

**Visualization:** Liang Li.

**Writing – original draft:** Bing Xue.

**Writing – review & editing:** Bing Xue, Shanshan Ma.
